# Comparative Study of Senescent Th Biomarkers in Healthy Donors and Early Arthritis Patients. Analysis of VPAC Receptors and Their Influence

**DOI:** 10.3390/cells9122592

**Published:** 2020-12-04

**Authors:** Raúl Villanueva-Romero, Amalia Lamana, Marissa Flores-Santamaría, Mar Carrión, Selene Pérez-García, Ana Triguero-Martínez, Eva Tomero, Gabriel Criado, José L. Pablos, Isidoro González-Álvaro, Carmen Martínez, Yasmina Juarranz, Rosa P. Gomariz, Irene Gutiérrez-Cañas

**Affiliations:** 1 Departamento de Biología Celular, Facultad de Biología y Medicina, Instituto de Investigación Sanitaria Hospital 12 de Octubre (imas12), Universidad Complutense de Madrid, 28040 Madrid, Spain; ravillan@ucm.es (R.V.-R.); amaliala@ucm.es (A.L.); marissaf@ucm.es (M.F.-S.); macarrio@ucm.es (M.C.); selene@ucm.es (S.P.-G.); cmmora@bio.ucm.es (C.M.); gomariz@ucm.es (R.P.G.); irgutier@ucm.es (I.G.-C.); 2 Servicio de Reumatología, Instituto de Investigación Sanitaria Hospital La Princesa (IIS-IP), 28006 Madrid, Spain; ana6n92@gmail.com (A.T.-M.); tomeroeva@yahoo.es (E.T.); isidoro.ga@ser.es (I.G.-Á.); 3 Servicio de Reumatología, Instituto de Investigación Sanitaria Hospital 12 de Octubre (i+12), 28041 Madrid, Spain; gcriado@h12o.es (G.C.); jlpablos@h12o.es (J.L.P.)

**Keywords:** senescent Th cells, CD4^+^CD28^−^ T cells, VPAC receptors, VIP, early arthritis, rheumatoid arthritis

## Abstract

Pro-inflammatory CD4^+^CD28^−^ T cells are characteristic of immunosenescence, but also of several autoimmune/inflammatory diseases. Vasoactive intestinal peptide (VIP) acts as an anti-inflammatory and immunomodulatory mediator on these cells. Our objective was to study the mutual influence between senescent Th cells and VIP axis in early arthritis (EA), comparing with non-EA donors. We characterized the correlation between senescent Th cells and clinic parameters of EA as well as the behavior of senescent Th biomarkers by real-time PCR. Clinical data were systematically recorded at baseline and after 6 months of follow-up. The number of CD4^+^CD28^−^ T cells measured by sorting is higher in patients who initially meet ACR classification criteria for rheumatoid arthritis (RA) compared to those who were classified as undifferentiated arthritis (UA). A slight positive correlation between EA CD4^+^CD28^−^ T cells and CRP or ESR and a negative correlation with bone mineral density were found. Th senescent biomarkers in EA CD4^+^CD28^−^ T cells were similar to donors, however some of them increased after 6 months of follow-up. VPAC receptors were analyzed by real-time PCR and immunofluorescence, and CD4^+^CD28^−^ T cells showed higher expression of VPAC_2_ and lower of VPAC_1_, VPAC_2_ showing a significant increased expression in EA cells. Sorted CD4^+^CD28^−^ T cells were in vitro expanded in presence of VIP, wherein VIP increased senescent biomarker CD27, while it diminished CD57 or NKG2 senescent biomarkers. Our study demonstrates for the first time the existence of a link between senescent Th cells and the VIP axis.

## 1. Introduction

Immunosenescence includes a number of functional changes in the immune system traditionally derived from cellular aging, which cause a near-constant state of inflammation, decreased immunity against pathogens, deficient responses to vaccines, and an increased risk of autoimmunity [1,2]. Senescence profile of CD4 T cells, crucial cells in the development and functioning of immune response, is characterized by an expression loss of costimulatory molecules CD27 and CD28. This phenotypic hallmark is accompanied with accumulation of regulatory T cells (Treg), severe proliferation impairment, a decline of telomerase activity, with the subsequent shortening of the telomere length, and the rise of natural killer (NK) surface molecules like CD57 or inhibitory receptors like PD-1 [3,4]. Furthermore, they have been defined as cytotoxic T helper (Th)-1 type cells, due to the production of pro-inflammatory mediators like IFN-γ and TNF-α and cytotoxic molecules like perforin and granzyme B [5].

Early immunosenescence processes have been noticed under pathological conditions and cellular stress derived from chronic inflammatory diseases, infections, or immune-mediated disorders, such as rheumatoid arthritis (RA) [5,6,7]. Precisely, CD4^+^CD28^−^ T cells were first identified and characterized in patients with RA, where its frequency is positively correlated with advanced joint destruction and the development of extraarticular manifestations [8,9,10]. In this sense, some authors considered these cells as a prognostic marker of radiographic progression in early arthritis (EA) [8]. Indeed, different therapeutic approaches for RA as infliximab or abatacept are able to decrease this subpopulation of lymphocytes, through different mechanisms [11,12]. Several studies in patients with a final diagnosis of RA have revealed the key role of CD4^+^CD28^−^ T cells in the onset of this disease. These cells were positive for granzyme B expression and secreted IFNγ after in vitro stimulation [13]. Moreover, CD4^+^CD28^−^ T cells from RA synovial fluid produce IL-17, actively contributing to the perpetuation of joint inflammation [14]. Recently, Fessler and collaborators have described a novel CD28^−^ Treg-like cells that show weak suppression capabilities on the proliferation of effector Th cells [15]. Besides, CD4^+^CD28^−^ T cells have a role in bone erosion, promoting osteoclastogenesis through a high RANKL production [16]. 

The cellular microenvironment has a main role during the immunosenescence process, as different factors influence cellular behavior and homeostasis. Such is the case with neuroendocrine-immune interactions that play a key role in this process [17]. An example of this interaction is the effect of the endogenous neuropeptide vasoactive intestinal peptide (VIP), secreted by nerve endings, endocrine, and immune cells, on lymphocytes or articular synoviocytes. This interaction establishes an important neuroimmune network in joints and humoral homeostasis during systemic or local inflammation and autoimmune responses, modulating both innate and adaptive responses [18,19,20]. This neuroimmunopeptide modulates the pathogenic activity of diverse cell subpopulations involved in RA, such as lymphocytes, synovial fibroblasts (SF), or macrophages. In this way, it modulates all the stages involved in the immune response, between the arrival of pathogens to the Th cell differentiation also observed in this pathology through its known anti-inflammatory and immunomodulatory actions. VIP is involved in this broad range of functions through its binding to its specific G-protein-coupled receptors, VPAC_1_ and VPAC_2_, that belong to B1 family of GPCRs [21,22,23]. The expression of these receptors changes with the type, state of differentiation, or activation of the cells. In this sense, the pattern of expression and cellular location of VPAC change in Th lymphocytes after their activation in healthy donors and EA patients [24]. From a therapeutic point of view, VIP could be used in two ways, on the one hand, as a therapeutic agent, given its proven anti-inflammatory and immunomodulatory potential, and on the other, together with its receptors, as biomarkers for personalized treatments in autoimmune/inflammatory diseases [18,19]. In this sense, we have described that genetic polymorphisms associated with high serum VIP levels are markers of low disease severity and lower therapeutic requirements. Therefore, this information could be useful to stratify patients for tailoring therapeutic effort during the “window of opportunity” period in EA patients [25,26]. In addition, lower levels of its receptor VPAC_1_ in peripheral blood mononuclear cells (PBMCs), are associated with more severe inflammation and increased disease activity in RA patients [27]. 

Taking into account all the abovementioned, the aims of the present study were to comparatively analyze the senescent Th biomarkers in healthy donors and EA patients and to examine the bidirectional influence between the VIP axis and senescent Th cells. We have found differences in markers of immunosenescence between donors and EA patients and changes associated with the evolution of the disease. Moreover, we have shown different expression pattern of VIP receptors between senescent and nonsenescent cells and the effect of this neuropeptide on senescent markers.

## 2. Materials and Methods

### 2.1. Study Population

Twenty-five patients (Table 1) belonging to the Princesa Early Arthritis Register Longitudinal (PEARL) study were analyzed. PEARL study was approved by Ethics Committees of Hospital Universitario La Princesa (Madrid, Spain; PI-518). All patients signed an informed consent form before data were included in the register, and biological samples were stored at the local Biobank. The PEARL study includes patients with more than 1 swollen joint for at least 4 weeks and symptoms for less than a year. Baseline blood samples were collected before treatment prescription. The register protocol includes collection of sociodemographic, clinical, and therapeutic data, as well as samples at each of the several scheduled visit. For this study, we have checked baseline and 6 months of follow-up. 

The initial diagnosis of the patients included in the study was distributed in 24% RA, 52% undifferenced arthritis (UA), and 24% arthralgia suspicious for progression to rheumatoid arthritis (clinically suspect arthralgia, CSA) (Table 2).

Peripheral blood samples from 13 healthy donors (non-EA donors) were included in this study (Table 1). The study was performed according to the recommendations of the Declaration of Helsinki and following the Spanish Personal Data Protection law, the patients’ demographic information was confidential. All donors signed an informed consent form before sampling. Samples and data from patients included in this study were provided by the Biobank Biobanco Hospital Universitario de La Princesa (ISCIII B.0000763), and they were processed following standard operating procedures with the appropriate approval of the Ethics and Scientific Committees.

### 2.2. BMD Measurements

Bone mineral density (BMD) was assessed using dual-energy X-ray absorptiometry (DXA) on a Hologic© QDR-4500 Elite (Bedford, MA, USA) at lumbar spine (LS) and hip. Furthermore, in 2004, we started to scan BMD at nondominant hand to study the effect of joint swelling on juxta-articular bone mass. Specifically, we analyzed BMD from L2 to L4, total hip (TH), and femoral neck (FN), and at hand, we assessed BMD from second to fifth MCP joints, as previously described [28]. BMD is expressed in grams per square centimeter.

### 2.3. Isolation of CD4^+^ T Cells Subsets

PBMCs were isolated from both EA patients and non-EA donors peripheral blood samples by density gradient (Ficoll-Hypaque, Sigma Aldrich, Merck, Darmstadt, Germany). CD4+ T-cells subsets (CD28^+^ and CD28^−^) were isolated from PBMCs by sorting (BD Influx), using antibodies against surface molecules CD3 (FITC), CD4 (APC), and CD28 (PE) (BD, Biosciences). Postsort analysis was performed for purity validation (≥95% at least) of selected cells. Autofluorescence and isotype controls were set up to determine the nonspecific fluorescence signal.

### 2.4. RNA Extraction and Semiquantitative Real-Time PCR

RNA extraction was performed using MicroElute Total RNA Kit (Omega Bio-tek). RNA was reverse-transcribed using High Capacity cDNA Reverse Transcription Kit (Applied Biosystems, Thermo Fisher Scientific, Madrid, Spain). CD27, hTERC, CDKN2A (p16), PD1, KLRC4, B3GAT1 (CD57), and RANKL were tested by Semiquantitative RT-PCR analysis using TaqMan Gene Expression technology (Applied Biosystems) and normalized with succinate dehydrogenase complex (SDHA) by the formula 2^−ΔCt^ (Table 3). Amplification was performed in a QuantStudio 7 Flex thermocycler (Applied Biosystems). VIP receptors (VIPR1 and VIPR2) were tested by semiquantitative RT-PCR using RealTime Ready Assay probes (Roche Life Science, Barcelona, Spain) and normalized with Glyceraldehyde-3-phosphate dehydrogenase (GAPDH), using again the formula 2^−ΔCt^ (Table 3). Amplification was performed in a LightCycler^®^ 480 Instrument II (Roche Life Science).

### 2.5. Immunocytochemistry Staining

Cell suspensions were seeded in PBS 1x and hold onto SuperFrost Plus Slides (Thermo Fisher Scientific, Madrid, Spain) for 30 min at 37 °C, 5% CO_2_. Then slides were washed with PBS 1x, fixed, and permeabilized. After rehydration and blocking, slides were incubated with rabbit polyclonal antihuman VPAC_1_ antibody (1:100, Thermo Fisher Scientific) and mouse monoclonal antihuman VPAC_2_ (1:50, Abnova, AntibodyBcn, Barcelona, Spain) 1 h at RT. After washing, Alexa Fluor 488 donkey antirabbit IgG and Alexa Fluor 594 goat antimouse IgG (1:500, Invitrogen, Thermo Fisher Scientific, Madrid, Spain) were used as secondary antibodies (1 h at RT). Counterstaining was performed with 1 mg/mL Hoechst. Negative controls were performed in the absence of anti-VPAC_1_ and anti-VPAC_2_ antibodies. Fluorescence was examined on a Leica SP-2 Acousto-Optical Beam Splitter confocal microscope with inverted stand (Leica DM IRE2; objective, 63X; Leica, L’Hospitalet de Llobregat, Spain). Images were analyzed by ImageJ (https://imagej.net/Fiji).

### 2.6. In Vitro Expansion of CD4^+^CD28^−^ T Cells

CD4^+^CD28^−^ T cells from 5 non-EA donors were cultured separately in 96 U-wells plates (0.1 × 10^6^ cells/well) with ImmunoCult-XF T Cell Expansion Medium (Stem Cell tech., Grenoble, France) supplemented with 1% penicillin/streptomycin (Gibco, Thermo Fisher Scientific, Madrid, Spain). Plates were precoated with 2 µg/mL of α-CD3 antibody (Clone OKT-3, BioLegend, Palex Medical, Barcelona, Spain) diluted in phosphate-buffered saline, in order to activate/expand T cells. Cells were cultured in the absence or presence of VIP 10 nM (Bachem A.G., Bubendrof, Switzerland) for 7 days.

### 2.7. Statistical Analysis

Pearson’s coefficient test for correlation analysis was performed using STATA software (StataCorp). Wilcoxon test for paired samples and Mann–Whitney test for unpaired samples were performed using GraphPad Prism 8 software (GraphPad Software, San Diego, CA, USA). 

## 3. Results

### 3.1. Frequency of Resting CD4^+^CD28^−^ Cells in Non-EA Donors and EA Patients.

**Figure 1:**
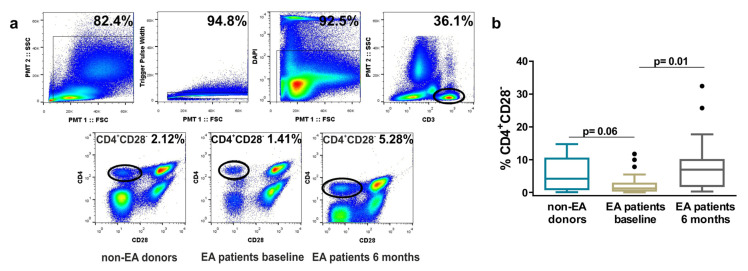
Frequency of resting CD4^+^CD28^−^ cells in non-early arthritis (non-EA) donors and EA patients determined by flow cytometry. (**a**) Up, representative gates to identify CD3^+^ cells are shown. Down, a representative dot plot analysis in each study group is shown. (**b**) The percentage of CD4^+^CD28^−^ cells from 13 non-EA donors and 25 EA patients at baseline and after 6 months of disease progression is shown. Data are presented as the interquartile range (p75 upper edge of the box, p25 lower edge, and p50 midline), p90 (line above the box), and p10 (line below the box). Dots represent outliers. Statistical significance was established using Mann–Whitney (non-EA/EA baseline) and Wilcoxon *t* test (EA baseline/EA after 6 months).

We studied the differences in the percentage of these cells between non-EA donors and EA patients, at baseline and after 6 months of disease progression. As this percentage could be conditioned by age, we checked the age of donors and EA patients, finding significant differences between both groups (Table 1). However, although donors showed a slight increase in the percentage of these cells with respect to EA patients at baseline, the differences were not significant (Figure 1). These patients are those first-time visiting the rheumatology consultation with more than 1 swollen joint for at least 4 weeks and symptoms for less than a year, without receiving a specific treatment for the pathology. However, after 6 months of disease progression, the percentage of CD4^+^CD28^−^ cells increased significantly with respect to baseline. 

### 3.2. Correlation between the Percentage of Resting CD4^+^CD28^−^ Cells and Clinical, Radiological, and Analytical Variables in EA Patients

**Figure 2:**
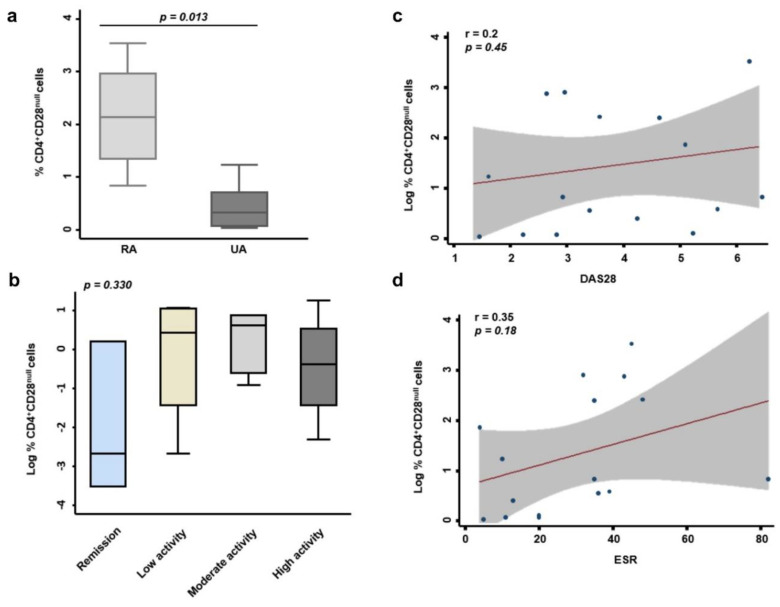
Correlation between the percentage of resting CD4^+^CD28^−^ cells and clinical, radiological, and analytical variables in EA patients. (**a**) Percentage of CD4^+^CD28^−^ cells according to initial diagnosis is shown. Graph box represents *n* = 4 patients with rheumatoid arthritis (RA) and *n*= 8 patients with undifferentiated arthritis (UA). Statistical significance was determined with Mann–Whitney test. Significance threshold was set at *p* < 0.05. (**b**) Logarithmic transformation of the percentage of CD4^+^CD28^−^ cells according to the degree of disease activity is shown. The activity of the disease in EA patients at baseline was classified according to 28-joint disease activity score (DAS28) into remission (<2.5), low activity (2.5–5.0), moderate activity (5.0–7.5), and high activity (7.5–10). Correlation between the percentage of these cells and DAS28 (**c**) and erythrocyte sedimentation rate (ESR) (**d**) was determined by the use of Pearson’s coefficient test. Data from 20 EA patients at baseline were analyzed.

We analyzed by sorting studies the percentage of CD4^+^CD28^−^ cells in patients with EA, and its correlation with clinical, radiological, and analytical variables in these patients. Our study population is heterogeneous, since we have 24% of patients who were classified as RA in the initial diagnosis, but there are 52% of undifferentiated arthritis (UA) and 24% of clinically suspect arthralgia (CSA) (Table 2). To analyze the percentage of CD4^+^CD28^−^ cells in this study population, we must take into account that UA groups patients who cannot yet be classified within a specific diagnosis according to the classification criteria of the American College of Rheumatology (ACR) and that patients classified as CSA are in an earlier stage and only show joint pain. Our data show that the percentage of CD4^+^CD28^−^ cells is significantly higher in patients who meet ACR classification criteria compared to UA (*p* = 0.013, Figure 2a). For this analysis, we decided to exclude patients classified as CSA, due to their high variability.

No significant correlation between the percentage of senescent cells and the activity index “disease activity score 28” (DAS28) or “Hospital Universitario Princesa Index” (HUPI) were observed (Figure 2 and data not shown). Nonetheless, taking into account the level of disease activity (remission, low, moderate, or high) assessed through DAS28, the percentage of CD4^+^CD28^−^ was lesser in remission EA patients (Figure 1). There were also no differences between positive or negative anticitrullinated protein antibody (ACAP) patients, nor positive or negative rheumatoid factor (RF) patients (data not shown). However, there was tendency to a positive correlation between the percentage of these cells and some analytical variables such as erythrocyte sedimentation rate (ESR) and C-reactive protein (CRP) (Figure 2 and data not shown). 

### 3.3. Characterization of Senescent Biomarkers in Resting CD4^+^CD28^−^ Cells from Non-EA Donors and EA Patients

The expression of CD28 was checked in the subpopulations CD4^+^CD28^+^ and CD4^+^C28^−^ obtained after cell separation by flow cytometry. It was proved that there was no expression of CD28 in the isolated CD4^+^CD28^−^cells, neither at RNA nor at protein level (data not shown). CD4^+^CD28^−^cells are characterized by the increase or decrease in the expression of several molecules as phenotypic surface markers like CD27, CD57, programmed cell death 1 (PD1), or the NK group 2-member D (NKG2D) or telomerase activity and cell cycle markers such as human telomerase RNA component (hTERC) or p16 [5,29].

**Figure 3:**
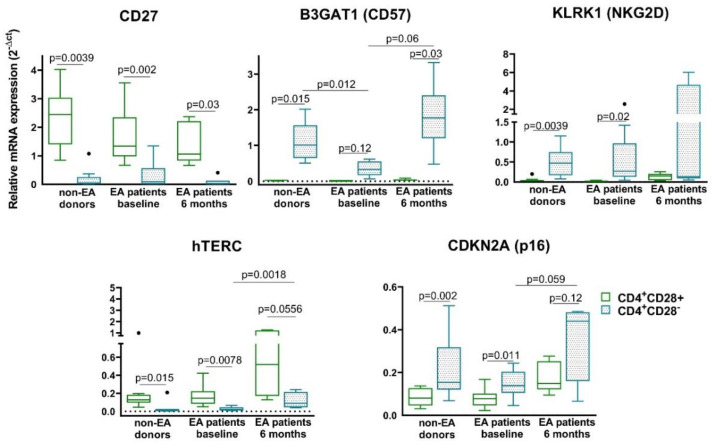
Characterization of senescent biomarkers in resting CD4^+^CD28^−^ cells from non-EA donors and EA patients. mRNA expression of CD27, β-1,3-glucuronyltransferase 1 (B3GAT1) (CD57), killer cell lectin like receptor K1 (KLRK1) (NKG2D), human telomerase RNA component (hTERC), and cyclin-dependent kinase inhibitor 2A (CDKN2A) (p16) was determined by semiquantitative real-time PCR analysis in non-EA donors, EA patients at baseline, and after 6 months of disease progression. Results are expressed as relative mRNA levels (normalized to succinate dehydrogenase complex flavoprotein subunit A (SDHA) mRNA levels, 2^−ΔCt^). Data are presented as the interquartile range (p75 upper edge of the box, p25 lower edge, and p50 midline), p90 (line above the box), and p10 (line below the box) from 11 different non-EA donors and 12 EA patients. Dots represent outliers. Statistical significance was established using Wilcoxon *t* test for the comparison between CD4^+^CD28^+^ and CD4^+^CD28^−^ and Mann–Whitney test for the comparison between cells from different groups.

In addition to the loss of CD28, Th senescent cells from the three groups under consideration were characterized by the loss of costimulatory molecule CD27 and also the telomerase activity measured by hTERC gene expression. As expected, the other senescent biomarkers tested suffered an increase in their mRNA expression in CD4^+^CD28^−^ T cells (Figure 3). The mRNA expression of CD57 in CD4^+^CD28^−^ T cells was the only one that showed differences between donors and EA patients at baseline, being its expression smaller in CD4^+^CD28^−^ T cells from EA patients than in those from non-EA donors. Moreover, we observed an increase in the mRNA expression of CD57, hTERC, and p16 in CD4^+^CD28^−^ T cells in EA patients after 6 months of disease progression. We also measured PD-1, a molecule characteristic of T cell exhaustion, because it has been associated with replicative senescence as well [30,31]. The profile of PD-1 was similar to CD57, NKG2D, or p16, with an increase in CD4^+^CD28^−^ T cells with respect to their counterpart CD28^+^ (data not shown) and no differences were observed according to CD4^+^CD28^−^ T cells coming from patients or donors.

In summary, no major differences were observed in the expression of the senescent biomarkers tested between non-EA donors and EA patients at baseline in CD4^+^CD28^−^ T cells. On the contrary, an increasing trend in the expression of some markers such as CD57, NKG2D, p16, or hTERC was observed in patients after 6 months of disease progression.

### 3.4. Relationship between Resting CD4^+^CD28^−^ Cells and Bone Loss.

**Figure 4:**
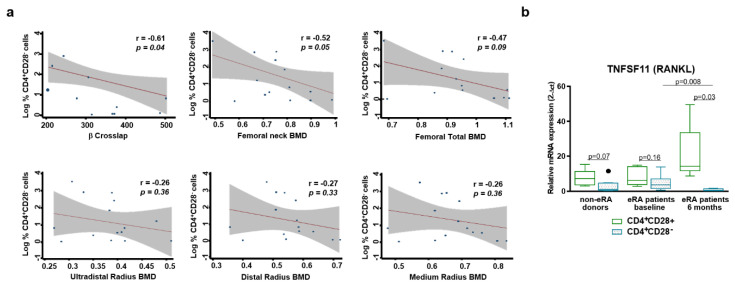
Relationship between resting CD4^+^CD28^−^ cells and bone loss. (**a**) Correlation between the percentage of these cells and β-Crosslap, Bone Mineral Density( BMD) total femur, BMD neck femur, and BMD distal radium was determined (Section 2.2, Materials and Methods). EA patients at baseline were analyzed. (**b**) mRNA expression of receptor activator of nuclear factor-kappa B (RANKL) was determined by semiquantitative real-time PCR analysis in non-EA donors, EA patients at baseline, and after 6 months of disease progression. Results are expressed as relative mRNA levels (normalized to SDHA mRNA levels, 2^−ΔCt^). Data are presented as the interquartile range (p75 upper edge of the box, p25 lower edge, and p50 midline), p90 (line above the box), and p10 (line below the box) from 8 different non-EA donors and 10 EA patients. Dots represent outliers. Statistical significance was established using Wilcoxon *t* test for the comparison between CD4^+^CD28^+^ and CD4^+^CD28^−^ and Mann–Whitney test for the comparison between cells from different groups.

It has been previously described that senescent Th cells promote bone loss in RA patients [16], therefore, we checked this fact in our EA patients, by testing the levels of alkaline phosphatase and β-Crosslap in the serum of patients and their bone mineral density measured by dual X-ray absorptiometry (DXA). While levels of alkaline phosphatase, which is a variable influenced by several factors, did not show any correlation with the percentage of CD4^+^CD28^−^cells, the levels of β-Crosslaps of type I collagen containing cross-linked C-telopeptide (β-CTX), a bone turnover marker, showed a negative correlation (Figure 4a and data not shown). In addition, it was also observed tendency to a negative correlation of these cells’ percentage with BMD of femur and radium from EA patients at baseline. Then, we checked the mRNA expression of RANKL, an important mediator of bone loss, in resting CD4^+^CD28^−^ T cells from non-EA donors and EA patients. We detected a decrease in the mRNA expression of these cells comparing with their CD4^+^CD28^+^ counterpart (Figure 4b). Besides, a significant decrease was observed in the expression of RANKL in resting CD4^+^CD28^−^ T cells from EA patients after 6 months of disease progression compared to baseline, whereas there is an increase in its expression in CD28^+^ cells. In summary, taking into account that BMD is influenced by several factors, such as age, sex, etc., we observed a tendency to a negative correlation between the percentage of CD4^+^CD28^−^ cells and the BMD in EA patients. Moreover, these data do not correspond to a higher RANKL expression in these cells in a nonactivated or resting state.

### 3.5. Expression of VPAC_1_ and VPAC_2_ in Resting CD4^+^CD28^−^ Cells from Non-EA Donors and EA Patients

**Figure 5:**
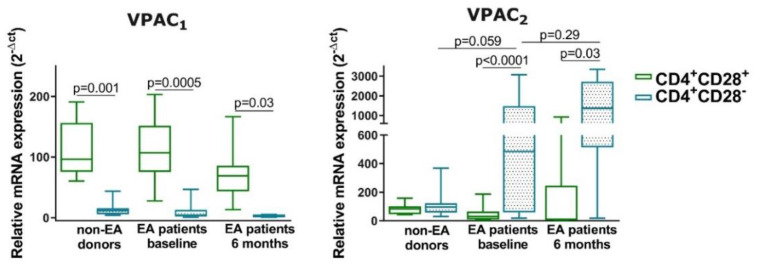
mRNA expression of VPAC_1_ and VPAC_2_ in resting CD4^+^CD28^−^ cells from non-EA donors and EA patients. RNA expression of VPAC_1_ and VPAC_2_ receptors was determined by semiquantitative real-time PCR analysis in non-EA donors, EA patients at baseline, and after 6 months of disease progression. Results are expressed as relative mRNA levels (normalized to GAPDH mRNA levels, 2^−ΔCt^). Data are presented as the interquartile range (p75 upper edge of the box, p25 lower edge, and p50 midline), p90 (line above the box), and p10 (line below the box) from 11 different non-EA donors and 12 EA patients. Statistical significance was established using Wilcoxon *t* test for the comparison between CD4^+^CD28^+^ and CD4^+^CD28^−^ and Mann–Whitney test for the comparison between cells from different groups.

**Figure 6:**
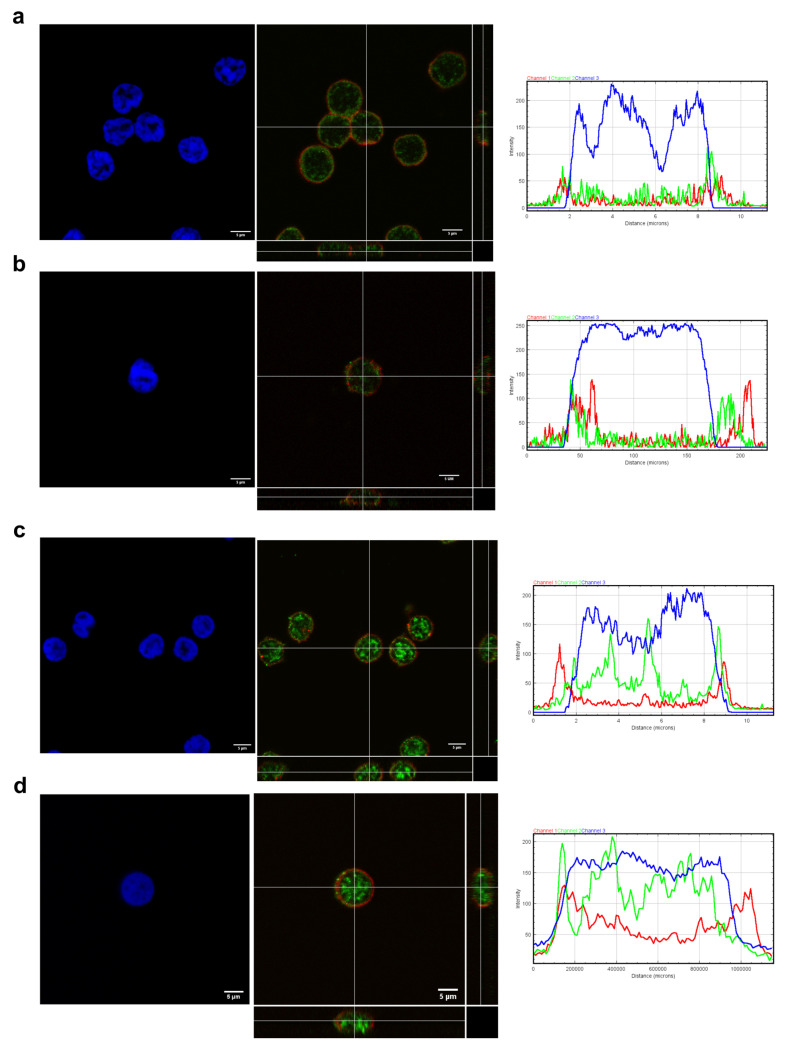
Cellular location of VPAC_1_ and VPAC_2_ in resting CD4^+^CD28^−^ cells from non-EA donors and EA patients. Immunofluorescence analysis showing the distribution of VPAC receptors across the cells ((**a**) non-EA donors CD4^+^CD28^+^, (**b**) non-EA donors CD4^+^CD28^−^, (**c**) EA donors CD4^+^CD28^+^, and (**d**) EA donors CD4^+^CD28^−^). Zoomed 63x images were imported into ImageJ to analyze the distribution of VIP receptors across the cells. Representative graphs of intensity profiles plotted as a function of distance (measured in pixels) versus intensity (measured in RGB-scale values) showed a distinctive distribution of receptors on each cell state. A representative experiment of three others is shown. Orthogonal projections of merged images are shown. In the lower side: XZ plane and in the right side: YZ plane. Images were obtained in a confocal microscope as explained in Materials and Methods. Scale bar represent 5 µm.

Different statuses of Th cells induce changes in the VPAC pattern expression and cellular location. We have previously shown that activated and differentiated lymphocytes showed different localization expression of these receptors, thus, we aimed to know if senescence also influenced their expression [22,24]. We observed in non-EA donors and in both EA patients at baseline and after 6 months of follow-up that resting CD4^+^CD28^−^ showed less mRNA expression of VPAC_1_ than their CD28^+^ counterparts while expression VPAC_2_ was augmented in these senescent cells (Figure 5). Both the loss and the gain were greater for EA patients than for non-EA donors, observing minor modifications between EA patients at baseline and after 6 months of disease progression. Regarding the distribution of these receptors in the cells on non-EA donors and EA patients at baseline by fluorescence intensity and orthogonal view, VPAC_1_ appeared both in plasma membrane and nuclear localization in resting CD4^+^CD28^−^ or CD4^+^CD28^+^ cells from both groups (Figure 6), whereas VPAC_2_ receptor is limited to plasma membrane in all cases studies. This effect is best observed in cells from EA patients, as these cells, although resting, are larger and the space between the plasma membrane and the nuclear membrane is greater than in the case of non-EA donors. Moreover, brighter staining of VPAC_1_ in the nucleus was observed in cells from EA patients. Thus, the senescence of Th cells induces a change in the pattern mRNA expression of VPAC receptors, without effect in their cellular location in a resting state.

### 3.6. Effect of VIP on Senescent Biomarkers in Non-EA Donors CD4^+^CD28^−^ Cells.

**Figure 7:**
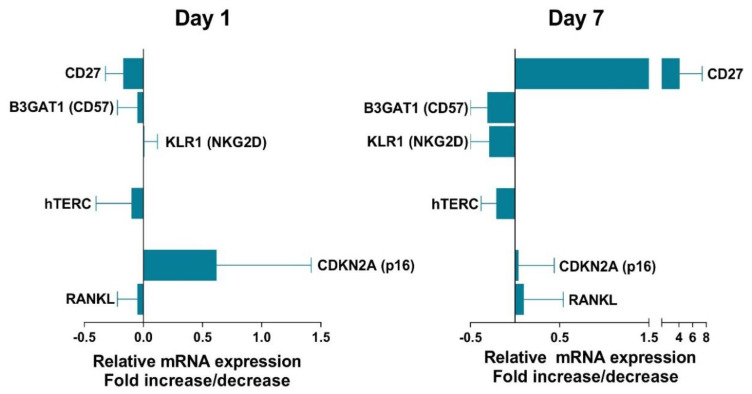
Effect of vasoactive intestinal peptide (VIP) on senescent biomarkers in non-EA donors CD4^+^CD28^−^ cells. mRNA expression of senescent biomarkers in non-EA donors CD4^+^CD28^−^ cells was tested in presence of 10 nM VIP after 1 or 7 day of cell culture activation. The fold change expression in the presence of VIP is represented. Data are the means ± SD of three different experiments performed in duplicate.

To analyze the influence of VIP on senescent Th cells, we performed in vitro studies of CD4^+^CD28^−^ cells from non-EA donors. The limitation of these studies was the low number of cells we could work with. We could only test the effect of VIP treatment after 1 and 7 days of cell activation, these periods represent a short and a long period for human Th cell activation. As we can observe in Figure 7, only a slight effect of VIP was detected in senescent biomarkers. CD27, a biomarker that decreases in CD4^+^CD28^−^ cells, was increased in the presence of VIP after 7 days of culture, whereas other biomarkers that increase in senescent T cells, such as CD57 or NKG2D, were decreased in presence of VIP. Another biomarker increased in senescent Th cells, p16, was weakly augmented in the presence of VIP at day 1, but not after 7 days of culture. Besides, no effect of VIP was observed on RANKL expression in these cells. Hence, although VIP effects were slight, they were much better observed after 7 days of culture with a tendency to induce a less senescent profile in CD4^+^CD28^−^ cells from non-EA donors.

## 4. Discussion

This study tries to evaluate, for the first time, the bidirectional influence between the senescence of Th cells and an anti-inflammatory and immunomodulatory axis, VIP and VPAC receptors. To perform it, we have used cells from EA patients, which showed an incipient anti-inflammatory/autoimmune disease, comparing them with those from non-EA donors. We have also done a deep scrutiny of the correlation between senescent Th cells and clinic parameters of EA as well as the behavior of senescent Th biomarkers during the disease. One limitation of this study is the low number of patients included, which prevent us to stratify them in order to improve the statistical significance. Nonetheless, it is the first study to describe these senescent cells in EA patients instead of patients with an established disease. 

In patients with RA, T-cell immunosenescence occurs prematurely and an accumulation of CD4^+^CD28^−^ cells is observed [9,10,14]. The study population in most cases were patients with an established disease. In some of them, they considered the differences between non-active RA and active RA, or the influence of some treatments, such as anti-TNFα or CD28 costimulation blocker, but only one of them analyzed this subpopulation in the early stages of the disease [8,11,12,32]. For these reasons, we have analyzed samples from the incipient state of the disease, early arthritis, and after 6 months of follow-up. It is important to bear in mind that the immunosenescence is not exclusive of one autoimmune disease, nor does it affect only T lymphocytes, in this way, it has been described expansion of these T cells in other pathologies, such as multiple sclerosis or systemic lupus erythematosus, and other cells of the innate immune system, such as dendritic cells, showed impairment of their functions in elderly people [33]. Moreover, in RA, immunosenescence can affect other cell types involved in this pathology, such as SF [5,6,34]. However, T cells seem to be a good model to study immunosenescence as the number of naïve T cells decreases with the age and memory T cells change their phenotype and their proportion, with an increased immunological risk when the ratio CD4/CD8 is inverted [35]. Therefore, expanded CD4^+^CD28^−^ cells can produce large amounts of pro-inflammatory cytokines and also have cytotoxic potential, which may cause tissue damage and development of pathogenesis in many inflammatory/autoimmune diseases [4,5]. 

Interestingly, in our study, the percentage of CD4^+^CD28^−^ is higher in patients who are classified as RA from the beginning, compared to those with UA. The number of patients available for this analysis is small and we also excluded patients with CSA, since this group introduced more variability, however these data are statistically significant. In addition, this result is consistent with previously published data in patients with established RA, where an accumulation of CD4^+^CD28^−^ cells is observed [9,10,14]. Furthermore, we observed a lower percentage of CD4^+^CD28^−^ cells in the group of patients classified in remission according to their DAS28 value when compared with those with moderate or high activity. The linear correlation between DAS28 and percentage CD4^+^CD28^−^ cells is moderate, however, there is also a trend towards positive correlation with other severity parameters, such as ESR or CRP.

In our study, numbers of circulating CD4^+^CD28^−^ T cells in baseline EA patients did not correlate with RA clinical parameters such as DAS28, HUPI, ACAP, or RF, according to previous results in patients with established RA [9,29]. Although the percentage of CD4^+^CD28^−^ T cells was not higher in baseline EA patients comparing with non-EA donors, the values increased significantly after 6 months of disease development in a similar way as mentioned above in more established periods of the disease. All this, despite the fact that the EA patients were significantly younger than the non-EA donors, could suggest that these patients develop a premature aging and promote an age-inappropriate expansion of CD4+CD28^−^ T cells, as other authors have described for several chronic inflammatory diseases [4]. In addition, it is important to bear in mind that if the percentage of senescent CD4 cells increased in peripheral blood after 6 months of disease development, over total of CD4 cells, the percentage of nonsenescent CD4 cells decreased. Another possible discrepancy observed is that, after 6 months of treatment, the patients decrease the DAS28, and in our study, we have observed a trend towards a positive correlation between the percentage of CD4^+^CD28^−^ cells and some analytical variables such as ESR and CRP. However, it seems that the increase in CD4^+^CD28^−^ cells observed at 6 months of follow-up is not directly related to disease activity (no correlation is observed between the variation in CD4^+^CD28^−^ cells between the baseline visit and the 6-month follow-up and DAS28 at each visit, data not shown). The explanation could be a reflection of the CD4 migration between the peripheral blood and the sites of inflammation, since CD4^+^CD28^−^ cells acquire their senescent profile in inflammatory sites. A greater presence of these cells in the peripheral blood of these patients after 6 months of treatment may be due to their migration from the sites of inflammation to the periphery.

Several studies demonstrate that the expansion of CD4^+^CD28^−^ cells is correlated with extraarticular manifestations and advanced joint destruction in RA [8,10,16], while others do not corroborate this fact [9]. Our results showed a tendency to negative correlation between the percentage of CD4^+^CD28^−^ cells and the femur BMD in baseline EA patients. This fact was not confirmed by alkaline phosphatase and β-CTX levels, being the first, a biomarker of bone formation and the second, a biomarker of bone resorption. It is not unexpected as our samples are from men and women with a broad range of age and these markers behave differently with sex and age; in men, markers of bone formation and resorption diminish when advancing age. However, in premenopausal women, they diminish with age, but in postmenopausal women, they are shown to be augmented [36]. We also tested RANKL expression in resting CD4^+^CD28^−^ T cells, and we detected a decrease in the mRNA expression of these cells compared with their CD4^+^CD28^+^ counterpart. Contrary to these results, studies by Fessler et al. describe a higher expression of RANKL in activated CD4^+^CD28^−^ T cells [16]. The difference between both studies may lie in the activation of the cells, since resting T cells negatively regulate osteoclast generation [37]. 

In addition to the lack of CD28 expression, loss of CD27 and telomerase length characterization have been studied in RA as biomarkers for senescent Th cells [32]. However, this is the first study to characterize other specific senescent biomarkers, such as CD57, PD1, NKG2D, hTERC, or p16 in CD4^+^CD28^−^ cells from early arthritis patients. CD4^+^CD28^−^ cells from EA patients are characterized, like those of the non-EA donors, by a loss in CD27, other costimulatory molecule, and in the telomerase activity measured by hTERC [5,29]. The expression of CD28 influences telomerase activity, thus CD4^+^CD28^−^ cells are characterized by shortened telomerase length [5,38]. As expected, the other senescent biomarkers tested, CD57, NKG2D, and p16, suffered an increase in their mRNA expression in CD4^+^CD28^−^ T cells with respect to their counterpart CD4^+^CD28^+^ cells. The mRNA expression of CD57 was the only one that showed differences between the senescent Th cells from EA patients and those of donors. CD57 (B3GAT1 gene) is currently being considered a marker of replicative senescence in Th lymphocytes, and it has been related in several cell types with a failure to proliferate while conserving the ability to produce cytokines upon encounter with their cognate antigen [39]. It is also important to highlight the differences in the expression of these markers after 6 months of disease follow-up. The mRNA expression of CD57, hTERC, and p16 in CD4^+^CD28^−^ T cells showed an increase with respect to those of baseline EA patients. The cell cycle inhibitor, p16 (CDKN2A), plays a major role in the senescence signaling program and an upregulation of this protein has been described in senescent CD4^+^ cells [29,40]. The slender higher expression of p16 in CD4^+^CD28^−^ T cells of donors compared to baseline EA patients could be due to the older age of non-EA donors, which is supported by the bigger expression in these cells of CD57, as it has been described that CD28^−^CD57^+^ T cells from older donors show higher expression of p16 than younger ones [41].

Once we have characterized and studied the possible correlation of senescent Th cells with EA pathology, we investigated the consequences that these cells have on the receptors of an important anti-inflammatory and immunomodulatory agent, VIP. The knowledge generated in animal models and in human ex vivo studies indicated that VIP and its signaling pathways could be used as a therapeutic agent and as a biomarker in inflammatory/autoimmune diseases. In humans, this neuroimmunopeptide controls the activation, polarization, and plasticity of Th cells in both healthy and pathological conditions [18,20,22,42,43,44,45]. It is important to dissect the pattern expression and cellular localization of VIP receptors, VPAC_1_ and VPAC_2_, to deep and better understand VIP effects on different states of CD4^+^ cells. As the senescence of Th cells has important consequences at several levels, we tried to determine if their response capacity to VIP also changes, modulating the expression of their receptors. Our results show that resting senescence of Th cells induces a change in the pattern of mRNA expression of VPAC receptors, without effect in cellular location in a resting state. Loss of mRNA expression of VPAC_1_ and gain of VPAC_2_ were greater in cells from EA patients. During the activation of human Th cells, the changes on mRNA expression of VPAC were similar to resting senescent Th cells, however, it does induce changes in VPAC cellular location [24]. Previous results showed the expression of VPAC_1_ and VPAC_2_ in PBMCs from EA patients during the clinical follow-up and reported that VPAC_1_ /VPAC2 mRNA ratio was lower in the early stages of the disease, moreover, the expression of VPAC_1_ increased considerably whereas the VPAC_2_ expression decreased after the 2-year follow-up [22,27]. Indeed, patients with more severe inflammation and higher disease activity show lower levels of VPAC_1_ expression, which is associated with patient-reported impairment. Thus, the VPAC_1_ loss observed in CD4^+^CD28^−^ T cells of these patients could be related with the fact that expanded senescent Th cells can produce large amounts of pro-inflammatory cytokines and also have cytotoxic potential in many inflammatory/autoimmune diseases [4,5]. In this sense, we show a correlation of senescent Th cells with some analytical disease markers and bone mineral density destruction. The changes in VPAC pattern expression in PBMCs from autoimmune diseases are not exclusives of EA patients. In PBMCs from patients with Graves’ disease, there is an increased expression of both receptors, however, only VPAC_2_ was functional in an adenylate cyclase-dependent signaling pathway [46]. Thus, changes in mRNA expressions of both receptors during different Th cell stages or different stages of an autoimmune disease such as EA suggest a possible dynamic regulation of both, probably acting as a compensatory mechanism. As for whether changes in the pattern of expression or cellular location of these receptors have functional consequences, in some cases, they do not vary and in other cases, they are different. For example, during the activation of human Th cells, both receptors exhibit a potent immunomodulatory capacity by controlling the pathogenic profile and the activation markers of Th cells [24], whereas VIP maintains a nonpathogenic profile during Th17 polarization and induces the upregulation of RORC, RORA, and IL-17 through VPAC_1_ and IL-23R or STAT3 through VPAC_2_ [45].

In addition of the important role of VIP during states of polarization, differentiation or activation from Th cells in healthy conditions or early arthritis conditions, our results show that it could also have consequences on the senescence of these cells, increasing the expression of CD27 and decreasing the expression of CD57 and NKG2D in of CD4^+^CD28^−^ T cells after 7 days of culture, trying to counteract the senescence in these cells. On the other hand, human senescent Th cells acquire a more pathogenic profile increasing the production IFNγ and IL-17 [5,14]. Therefore, the effect of VIP on human senescent cells may also be acting on this pathway, as VIP decreases the cytokine pathogenic profile of Th cells in both healthy donors and EA patients [43].

Overall, our study demonstrates for the first time the existence of a link between senescent Th cells and the VIP axis. There is an important mutual influence that could have significant consequences both from the point of view of understanding the mechanism of the pathology and from the therapeutic point of view.

## 5. Conclusions

- Patients who meet classification criteria for RA have a percentage of CD4^+^CD28^−^ higher than those with undifferentiated arthritis. In addition, patients who are classified in remission according to the disease activity defined by the DAS28 also present a percentage of CD4^+^CD28^−^ lower than those who present low, moderate, or high activity.
- Only a tendency to positive correlation between senescent Th cells and CRP or ESR and a negative correlation between these cells and bone mineral density were found in early arthritis patients.
- The expression of Th senescent biomarkers, CD27, CD57, PD1, NKG2D, hTERC, or p16, in senescent Th cells from early arthritis patients was similar to those of donors, however, some of them showed an increase after 6 months of disease progression.
- The senescence of Th cells induces a change in the mRNA pattern expression of VPAC receptors in a resting state, the loss of VPAC_1_ and gain of VPAC_2_ were greater in cells from early arthritis patients.
- VIP modulates some senescent Th biomarkers as CD27, CD57, or NKG2D, trying to counteract the senescence in these cells.

## Figures and Tables

**Table 1:** Characteristics of donors and baseline early arthritis.

	Non-EA Donors	EA Patients		Normal Range
		Baseline	6 Months	Normal Range
Number	13	25		
Age (years)	65.5 ± 16.5	51.6 ± 15.0 *		
Female gender, *n* (%)	5 (39%)	19 (76%)		
ACPA + (%)	n.d.	45		Negative: <20 U Grey zone: 20–39 U Positive: ≥40 U
RF + (%)	n.d.	52		0–14 U/mL
BMI	26.2 ± 3.57	26.4 ± 3.84		
DAS28	n.d.	3.92 ± 1.63	2.77 ± 1.35 ^#*p* = 0.016^	
HUPI	n.d.	5.38 ± 3.50	3.49 ± 2.87 *^p^*^= 0.06^	
ESR (mm/h)	n.d.	28.6 ± 19.4	21.2 ± 18.2	0–25 U
CRP (mg/dL)	n.d.	0.57 ± 0.53	0.29 ± 0.24 *^p^*^= 0.059^	0–0.5 mg/dL
AP (U/L)	n.d.	72.8 ± 16.1	71.6 ± 18.5	35–105 U/L
β-Crosslap (β-CTX)	n.d.	387 ± 133	379 ± 107	Pr.W. 137–573 pg/mL Po.W. 226–1008 pg/mL Men 30–50 y.o. 142–584 pg/mL Men 50–70 y.o. 200–704 pg/mL Men > 70 y.o. 230–854 pg/mL

Data are shown as the median ± standard deviation or *n* (%). ACPA: anticitrullinated peptide antibodies; RF: Rheumatoid factor; BMI: Body Mass Index; DAS28: 28-joint disease activity score; HUPI: Hospital Universitario Princesa Index; ESR: erythrocyte sedimentation rate; CRP: C-reactive protein; AP: alkaline phosphatase. n.d.: non determined; Pr.W.: premenopausal women; Po.W.: postmenopausal women; y.o.: years old. The asterisk character (*) shows the significant differences between non-EA donors and EA patients (*p* > 0.05), and pad character (#) shows the significant differences between EA-patients baseline and EA-patients after 6 months.

**Table 2:** Treatment and initial diagnoses of donors and early arthritis patients.

	Non-EA Donors	EA Patients
Treatment		
None, *n* (%)	13 (100)	14 (56)
MTX, *n* (%)	-	8 (32)
Antimalarial, *n* (%)	-	2 (8)
MTX + antimalarial, *n* (%)	-	1 (4)
Initial diagnosis		
RA, *n* (%)	-	6 (24)
UA, *n* (%)	-	13 (52)
CSA, *n* (%)	-	6 (24)

Data are shown as *n* (%). MTX: methotrexate; RA: rheumatoid arthritis; UA: undifferenciated arthritis; CSA: clinically suspicious arthralgias.

**Table 3:** Genes analyzed by semiquantitative real-time polymerase chain reaction using TaqMan Gene Expression technology (**A**) or RealTime Ready Assay probes from Roche (**B**). Gene, GenBank accession number and sequence or assay ID for each primer used in the study are shown.

(**A**)		
**Gene**	**GenBank Accession no.**	**Assay ID (TaqMan^®^)**
SDHA	NM_004168.3	Hs00188166_m1
CD27	NM_001242.4	Hs00154297_m1
hTERC	NR_001566.1	Hs03454202_s1
CDKN2A (p16)	NM_000077.4	Hs00923894_m1
PDCD1 (PD1)	NM_005018.2	Hs01550088_m1
KLRK1 (NKG2D)	NM_013431.2	Hs00255338_m1
B3GAT1 (CD57)	NM_018644.3	Hs01024500_m1
TNFSF11(RANKL)	NM_003701.3	Hs00243522_m1
(**B**)		
**Gene**	**GenBank Accession No.**	**Assay ID (Roche)**
GAPDH	NM_002046.7	141139
VIPR1 (VPAC_1_)	NM_004624.4	104081
VIPR2 (VPAC_2_)	NM_003382.5	148318

B3GAT1: β-1,3-glucuronyltransferase 1; CDKN2A: cyclin-dependent kinase inhibitor 2A; GAPDH: Glyceraldehyde-3-Phosphate Dehydrogenase; hTERC: human telomerase RNA component; KLRK1: killer cell lectin like receptor K1; NKG2D: natural killer group 2 member D; PDCD1: programmed cell death 1; RANKL: receptor activator of nuclear factor-kappa B ligand; SDHA: succinate dehydrogenase complex flavoprotein subunit A; TNFSF11: TNF Superfamily member 11. In the event that the name of the gene does not match the protein, it will appear in parentheses.
